# Transcatheter Versus Surgical Aortic Valve Replacement in Patients with Cardiac Surgery: Meta-Analysis and Systematic Review of the Literature

**DOI:** 10.3390/jcdd7030036

**Published:** 2020-09-10

**Authors:** Azka Latif, Noman Lateef, Muhammad Junaid Ahsan, Vikas Kapoor, Rana Mohammad Usman, Stephen Cooper, Venkata Andukuri, Mohsin Mirza, Muhammad Zubair Ashfaq, Rami Khouzam

**Affiliations:** 1 Department of Internal Medicine, Creighton University, Omaha, NE 68124, USA; noman.mlateef@gmail.com (N.L.); junaidahsan333@gmail.com (M.J.A.); VikasKapoor@creighton.edu (V.K.); StephenCooper@creighton.edu (S.C.); VenkataAndukuri@creighton.edu (V.A.); mohsin.mirza@creighton.edu (M.M.); Muhammad.Ashfaq@alegent.org (M.Z.A.); 2 Department of Internal Medicine, University of Tennessee, Memphis, TN 38152, USA; rusman@uthsc.edu; 3 Department of Cardiology, University of Tennessee, Memphis, TN 38152, USA; khouzamrami@yahoo.com

**Keywords:** surgical aortic valve replacement, transcatheter aortic valve replacement, TAVR, SAVR, prior CABG, redo-AVR, in-hospital outcomes, short-term outcomes

## Abstract

The number of patients with severe aortic stenosis (AS) and a history of prior cardiac surgery has increased. Prior cardiac surgery increases the risk of adverse outcomes in patients undergoing aortic valve replacement. To evaluate the impact of prior cardiac surgery on clinical endpoints in patients undergoing transcatheter aortic valve replacement (TAVR) versus surgical aortic valve replacement (SAVR), we performed a literature search using PubMed, Embase, Google Scholar, and Scopus databases. The clinical endpoints included in our study were 30-day mortality, 1–2-year mortality, acute kidney injury (AKI), bleeding, stroke, procedural time, and duration of hospital stay. Seven studies, which included a total of 8221 patients, were selected. Our study found that TAVR was associated with a lower incidence of stroke and bleeding complications. There was no significant difference in terms of AKI, 30-day all-cause mortality, and 1–2-year all-cause mortality between the two groups. The average procedure time and duration of hospital stay were 170 min less (*p* ≤ 0.01) and 3.6 days shorter (*p* < 0.01) in patients with TAVR, respectively. In patients with prior coronary artery bypass graft and severe AS, both TAVR and SAVR are reasonable options. However, TAVR may be associated with a lower incidence of complications like stroke and perioperative bleeding, in addition to a shorter length of stay.

## 1. Introduction

In the United States, degenerative aortic valve disease is the third most common cardiovascular disease affecting approximately 2.5 million adults [1,2]. Healthcare advancements have led to an increase in life expectancy, which has in turn increased the prevalence of elderly patients with aortic stenosis (AS). Medical management of severe AS has limitations such as a higher risk of disability and mortality, as well as a relatively low survival rate, approximately 50% over 2–3 years. Transcatheter aortic valve replacement (TAVR) is superior to medical management in patients with severe symptomatic AS in whom surgical aortic valve replacement (SAVR) is associated with a prohibitive risk [3]. With improving techniques, devices, and positive results from TAVR, its worldwide use is continually increasing. The SAVR, which is the current standard of care for severe AS, is more complex in the context of cardiac surgery. Redo coronary artery bypass graft (CABG) is associated with high morbidity and mortality ranging from 10–30% [4]. An increasing number of patients with severe AS have a history of prior cardiac surgery which is associated with increased risk of undergoing aortic valve replacement. In such cases, the proposed benefit of TAVR versus SAVR remains unknown. This study aimed to evaluate the impact of prior cardiac surgery on clinical endpoints in patients undergoing TAVR versus SAVR for severe AS.

## 2. Materials and Methods

**Figure 1:**
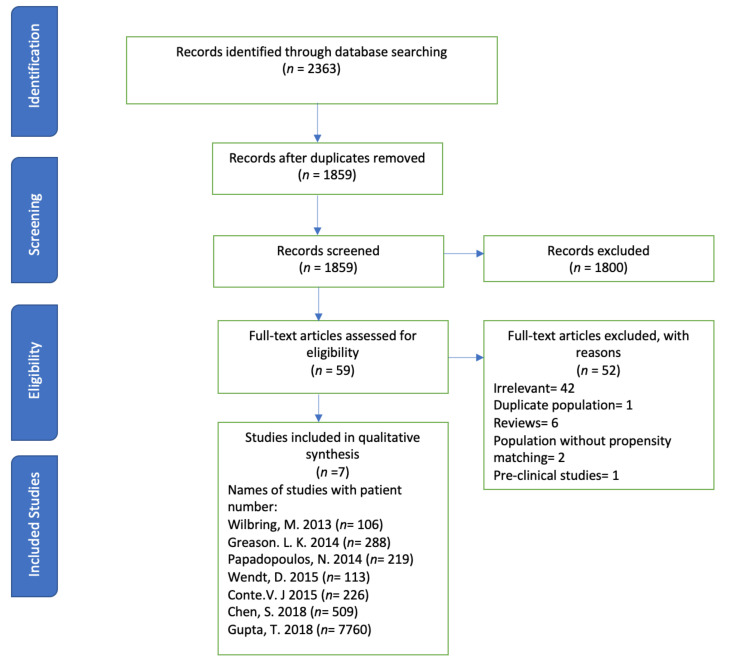
PRISMA flow showing the selection process of studies.

The systematic review was conducted according to the PRISMA statement and its summary is given in Figure 1.

A systematic search of databases such as PubMed, Embase, Web of Science, and Cochrane Library was performed using the medical search terms (MeSH) and their respective keywords with the following search strategy: “coronary artery bypass graft” AND “Aortic Valve Stenosis” AND “Transcatheter Aortic Valve Replacement”. Additionally, unpublished trials were identified from the clinicaltrials.gov website and references of all pertinent articles were also scrutinized to ensure the inclusion of all relevant studies. The search was completed on 21 January 2020 with no filters applied for language, subjects, or time period. After removing 504 duplicates, titles and abstracts of 1859 articles were screened for relevance by two independent reviewers, and conflict was settled by discussion. A total of 59 articles were deemed relevant and their abstracts and full texts were screened for eligibility. The following eligibility criteria were used: original articles reporting the most recent safety or efficacy outcomes for individual studies that comparatively evaluated the use of TAVR and SAVR in patients with any prior cardiac surgery.

Only seven studies qualified this strict selection criteria (number of patients (*n*) = 8221): all studies were retrospective. The reasons for exclusion for the other 52 articles were: duplicates (1), preclinical studies (1), irrelevance (42), reviews (6), and population without propensity matching (2). The exclusion criteria were preclinical studies, studies showing no subjective data regarding the safety or efficacy of SAVR/TAVR in patients with AS, studies including patients undergoing redo-SAVR or redo-AV replacement, and interim reports of studies other than the most recent one. The two studies were excluded although these studies included study population, but these observational studies reported outcomes without propensity matching of TAVR in AS. The included outcomes were 30-day all-cause mortality, 1–2-year all-cause mortality, procedural time, acute kidney injury (AKI), bleeding complications, stroke, and duration of hospital stay between TAVR and SAVR groups. The main summary estimate was random effects odds ratio (OR) with 95% confidence intervals (CIs). The seven studies satisfied the initial inclusion criteria.

The quality of the included studies and their risk of inherent bias was assessed using the NIH tool for quality assessment. The NIH tool for quality assessment evaluates an article based on the following variables: random sequence generation, allocation concealment, blinding of participants and personnel, blinding of outcome assessment, incomplete outcome data, selective reporting, and any other sources of bias. The bias risk assessment using this tool is mostly subjective. Two independent reviewers performed the risk assessment and the included studies showed a low risk.

## 3. Results

### 3.1. Baseline Characteristics

A total of seven studies (3 randomized trials, 4 cohort) including 9221 patients (4055 in the TAVR group and 4166 in the SAVR group) were included in the analysis [5,6,7,8,9,10,11]. Out of these, three were randomized control trials and four were prospective cohort studies. The mean age and percentage of male patients were 80 ± 6.6/78 ± 6.3 years and 75.5%/75.5% males in the TAVR and SAVR groups, respectively. Data were extracted on a pre-specified table (Table 1), which included the following parameters: 30-day mortality, 1–2-year mortality, post-operative stroke, major bleeding, mean length of hospital stay, discharge to home from the hospital, post-operative acute renal failure, and pacemaker implantation.

Wilbring, Wendt, Chen, and Papadopoulos et al. included patients with any prior cardiac surgery, while Greason, Conte, and Gupta et al. restricted their studies to patients with prior history of CABG. Wilbring, Papadopoulos, and Chen et al. included patients undergoing TAVR with a transapical approach. Wendt and Conte et al. included transapical as well as transfemoral approaches for TAVR. Chen et al. included transapical, transfemoral, and transaortic approaches, while Gupta et al. included endovascular and transapical approaches. Greason et al. did not mention about their approach. No evidence of publication bias was found. If desired data were not reported in a study, we documented them as not reported. Data were recorded as median or percentage.

### 3.2. All-Cause Mortality

Seven studies reported 361 cases of 30-day all-cause mortality in patients undergoing TAVR versus SAVR in previous cardiac surgery patients. There were 125 events out of 4055 (3.0%) that occurred in the TAVR group and 136 events out of 4166 (3.26%) that occurred in the SAVR group. There was no significant difference between the two groups (OR 0.87 [95% CI: 0.56–1.37], *p* = 0.54).

**Figure 2:**
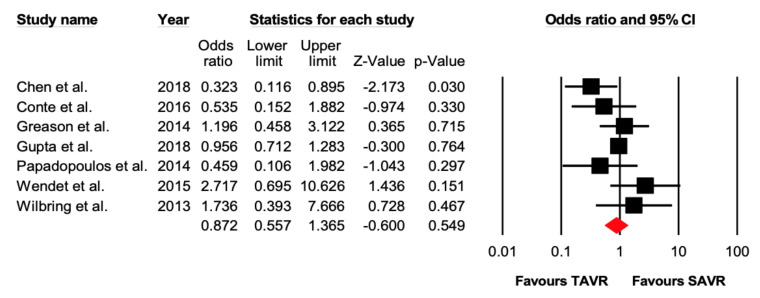
Forest plot comparing the risk of 30-day all-cause mortality between transcatheter versus surgical aortic valve replacement.

**Figure 3:**
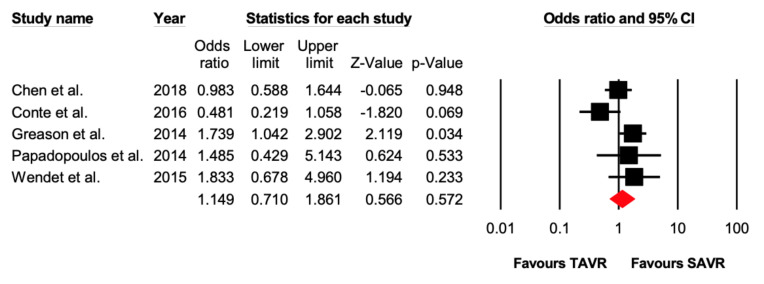
Forest plot comparing the risk of 1–2-year all-cause mortality between transcatheter versus surgical aortic valve replacement.

Five studies reported 1–2-year all-cause mortality. There were 126 out of 663 (19%) events that occurred in the TAVR group, while 108 out of 659 (16.4%) events occurred in the SAVR group. There was no significant difference (OR = 1.15 [95% CI: 0.71–1.86], *p* = 0.57) between the two groups (Figure 2 and Figure 3).

**Figure 4:**
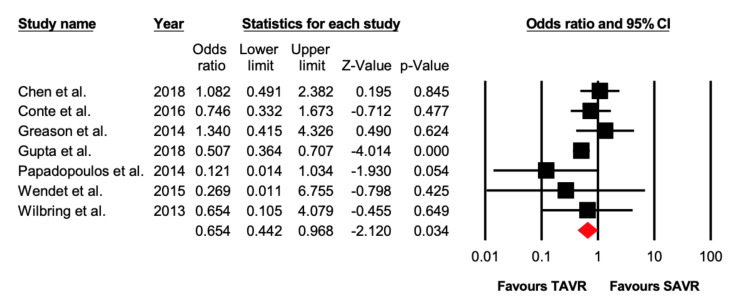
Forest plot comparing the risk of 30-day stroke between transcatheter versus surgical aortic valve replacement.

Seven studies reported data on stroke after the procedure. There were 89/4055 (2.19%) patients who suffered a post-operative stroke in the TAVR group, while 149/4166 (3.57%) patients had a stroke in the SAVR group. TAVR was associated with lower incidence of stroke (OR = 0.65 [95% CI: 0.44–0.97], *p* = 0.03), compared with SAVR in patients with previous cardiac surgery (Figure 4).

### 3.3. Post-Operative Bleeding

**Figure 5:**
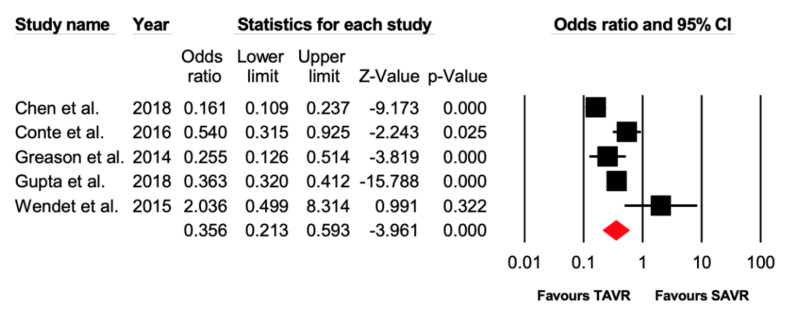
Forest plot comparing the risk of post-operative bleeding between transcatheter versus surgical aortic valve replacement.

Five studies reported data on the number of patients experiencing bleeding complications. There were 557/4055 (13.7%) patients in the TAVR group that had bleeding events, while 1252/4166 (30.0%) patients in the SAVR experienced bleeding events. TAVR was associated with lower incidence of bleeding complications (OR = 0.36 [95% CI: 0.21–0.59], *p* ≤ 0.01) compared with the SAVR group (Figure 5).

### 3.4. Acute Kidney Injury

Seven studies reported data on AKI. There were 699/4055 (17.2%) patients who suffered AKI in the TAVR group, while 860/4166 (20.6%) patients suffered AKI in the SAVR group. There was no statistical difference between both groups (OR = 0.71 [95% CI: 0.49–1.02], *p* = 0.06) (Supplementary Figure S1).

### 3.5. Procedure Time and Duration of Hospital Stay

Three studies reported data on the average procedure time and our study reported TAVR lasting 170 min less than SAVR (Mean difference = −170.95 [95% CI: −249.37, −92.53], *p* ≤ 0.01). Four studies reported the duration of hospital stay. TAVR was associated with shorter hospital stay (3.6 days [95% CI: −5.43, −1.95], *p* < 0.01) as compared to patients with SAVR, respectively (Supplementary Figure S2; Figure 3).

## 4. Discussion

Out meta-analysis study evaluated the outcomes of patients, undergoing TAVR versus SAVR, with a history of prior cardiac surgery. The main findings of our analysis were as follows: (1) no significant differences were seen in terms of 30-day and 1–2 years all-cause mortality between TAVR and SAVR groups. Two, there was no difference in post-operative AKI. Three, TAVR was associated with a lower incidence of stroke and post-operative bleeding. Four, the average procedural time was less in patients undergoing TAVR. Five, TAVR patients experienced early discharge from the hospital with a lower length of stay, compared to SAVR patients.

In patients with prior cardiac surgery, SAVR is challenging due to increased risk of bleeding, difficulties in myocardial perfusion, and injury to patent bypass grafts from adhesions. SAVR carries a mortality risk of 20% in high-risk patients [12]. TAVR emerged as an alternative therapy in patients with severe symptomatic AS who were deemed to be high or intermediate risk for open-heart surgery. Over the last decade, there has been an ongoing debate over the outcomes of TAVR versus SAVR in AS patients with prior history of cardiac surgery. The subgroup analysis of CABG patients in the placement of aortic transcatheter valves (PARTNER) trial reported high mortality risks with TAVR compared to SAVR [6]. These results were in contrast to the subgroup analysis of CABG patients in PARTNER-2 and US CoreValve High-Risk Study (CHR) [9]. PARTNER-2 trial demonstrated no significant difference in mortality between SAVR and TAVR at 1 year [13], while the CHR study showed that TAVR confers a mortality advantage over redo-SAVR. The results of PARTNER-2 trial are in accordance with the results of our meta-analysis. It is worth mentioning that first-generation balloon-expandable SAPIEN valves were used in the PARTNER trial, while newer generation SAPIEN XT valves were used in the PARTNER-2 trial. This explains better outcomes with newer generation devices and a possible explanation for the disparity in results between two trials. Operator experience could also be one of the elements which are evolving with time leading to improved outcomes with TAVR.

The risk of post-operative stroke was higher (3.8%) in TAVR when compared with SAVR (2.1%) in the PARTNER trial [6]. However, the subgroup analysis of CABG patients in CHR and PARTNER-2 trial revealed inverted results in terms of post-operative stroke [9,13], which correlates with our results (1.9% in TAVR group versus 3.3% in SAVR group). This might be explained by low risks of atrial fibrillation after TAVR when compared with SAVR (45% versus 20%) [14]. Furthermore, recent FDA approval of embolic protection devices in TAVR may have reduced the overall risk of stroke post-TAVR. Reinöhl et al. [15] reported a significantly higher stroke rate in patients with a previous history of CABG undergoing SAVR when compared with the ones without prior history of CABG.

Prior cardiac surgery increases the risk of bleeding during re-operative sternotomy due to injury of the heart or grafts from severe adhesions. In our analysis, the redo-SAVR group experienced more major bleeding compared with TAVR patients. These results are in concordance with the results of previous studies. Reinöhl et al. reported that the substantial risk of bleeding was two times more frequent in redo-SAVR patients [15]. Likewise, Nalluri et al. reported the risk of major bleeding in redo-TAVR versus redo-SAVR to be 25.1% and 9.1%, respectively (*p* ≤ 0.0001) [16].

There was no significant difference in regard to AKI in our analysis between SAVR and TAVR group. Some previous studies have shown a higher incidence of AKI among patients undergoing TAVR as compared to SAVR patients [17,18]. However, their results may have been confounded by selection bias, given the fact that TAVR patients are usually at a higher risk with an increased number of comorbid conditions, many of which are associated with a higher risk of post-procedural AKI. Furthermore, over the years, improvement in screening for the identification of patients with chronic kidney disease and minimal use of contrast with better imaging techniques, such as 3D transesophageal echocardiography and computed tomographic angiography, played an important role in reducing the incidence of AKI post-TAVR [19].

Resource utilization is among the secondary outcomes in favor of TAVR over SAVR. Our analysis showed shorter average procedural time and hospital stays in patients with TAVR compared to SAVR patients. Given that these patients are at high risk with significant other comorbid conditions, an early discharge to home instead of nursing homes is an additional benefit over SAVR for quick recovery.

Limitations of our study were as follows: First, this was a cohort study/trial-level meta-analysis as we did not have access to individual patient data and thus, the reason for individual decisions was unknown. Second, most of the studies included patients in the TAVR group who were deemed to be high risk with high STS-PROM (Society of thoracic surgeons-predicted risk of mortality) and EuroSCORE (European system for cardiac operative risk evaluation), putting them at higher risk for mortality and post-procedural complications. Finally, baseline characteristics were not similar in all included studies and the used access site for TAVR was not mentioned in all studies.

## 5. Conclusions

In patients with prior cardiac surgery and severe AS, both TAVR and SAVR are reasonable options. However, TAVR offers a fast and safe alternative for patients presenting with AS after previous cardiac surgery with a lower incidence of outcomes like stroke and perioperative bleeding, as well as a shorter length of stay. Future prospective studies can shed more light on this evolving and fast-growing field.

## Figures and Tables

**Table 1:** Characteristics of involved studies, and outcomes reported in patients who underwent transcatheter versus surgical aortic valve replacement with a history of prior redo coronary artery bypass graft (CABG).

Author	Age (years)		Male: Female		Number of Patients		30-Day Mortality Days (Percentage)		Post-Operative Stroke Days (Percentage)		1–2-Year Mortality Days (Percentage)		Mean Length of Hospital Stay (Days)		Acute Renal Failure Days (Percentage)		Procedural Time (Min)		Bleeding	
	TAVR	SAVR	TAVR	SAVR	TAVR	SAVR	TAVR	SAVR	TAVR	SAVR	TAVR	SAVR	TAVR	SAVR	TAVR	SAVR	TAVR	SAVR	TAVR	SAVR
**Wilbring, 2013**	78 ± 5.5	77.6 ± 2.7	26/27	35/18	53	53	5 (9.4)	3 (5.7)	2 (3.9)	3 (5.7)	9 (17)	7 (13.2)	12 ± 6.5	14.6 ± 8.7	50.8 ± 63.6	64.7 ± 67.6	47.9 ± 11.5	145.6 ± 33.8	NR	NR
**Greason, 2014**	80.7 ± 7	82.3 ± 6.2	120/28	111/29	148	140	10 (6.8)	8 (5.7)	7 (4.7)	5 (3.6)	90 (60)	59 (42.1)	NR	NR	6 (4.1)	6 (4.3)	NR	NR	12 (8.1)	36 (25.7)
**Wendt, 2015**	78.7 ± 5.9	71.1 ± 10.8	43/19	38/13	62	51	9 (14.5)	3 (5.8)	0	1 (2.0)	14 (22.6)	7 (13.7)	NR	NR	7 (11.3)	3 (5.9)	NR	NR	5 (8.0)	2 (3.9)
**Papadopoulos, 2014**	82 ± 5	72 ± 9	31/21	92/75	52	167	3 (6)	14 (8)	0	7 (4)	7 (13.4)	5 (2.99)	NR	NR	3 (5)	6 (4)	106 ± 53	332.5 ± 120	144 ± 209 ^a^ 2 (4) ^b^	580 ± 420 ^a^ 13 (8) ^b^
**Conte, 2016**	82 ± 5.8	81 ± 5.9	91/24	87/24	115	111	3.5 (4)	6.3 (7)	10.6 (12)	14.3 (15)	9.6 (11)	18.1 (20)	7.3 ± 5.7	11.8 ± 12.7	5.3 (6)	16.3 (18)	NR	NR	38 (33)	53 (48)
**Chen, 2018**	78.5 ± 7.0	79.4 ± 6.5	203/42	216/48	245	264	2.1 (5)	3.1 (8)	5.3 (13)	4.9 (13)	13.3 (32)	14.4 (35)	5.5 ± 3.0	10.5 ± 6.8	17.7 (43)	29.9 (77)	100.8 ± 46.5	294.0 ± 95.2	36.4 (89)	78.3 (206)
**Gupta, 2018**	80.7 ± 7.2	73.6 ± 8.7	6593/2292	5023/1147	3380	3380	186 (2.1)	154 (2.5)	142 (1.6)	166 (2.7)	NR	NR	6.5 ± 5.0	8.7 ± 5.9	1484 (16.7)	1098 (17.8)	NR	NR	1004 (11.3)	1487 (24.1)

^a^ Post-operative chest tube drainage (mL/24 h); ^b^ surgical re-exploration; Abbreviations: TAVR, transcatheter aortic valve replacement; SAVR, surgical aortic valve replacement; NR, not reported.

## Supplementary Materials

The following are available online at https://www.mdpi.com/2308-3425/7/3/36/s1, Figure S1: Forest plot comparing the risk of acute kidney injury between transcatheter versus surgical aortic valve replacement; Figure S2: Forest plot comparing the procedural time between transcatheter versus surgical aortic valve replacement.
