# Supplementary material for: Transcatheter Versus Surgical Aortic Valve Replacement in Patients with Cardiac Surgery: Meta-Analysis and Systematic Review of the Literature
Source: J Cardiovasc Dev Dis. 2020 Sep 10;7(3):36. doi: 10.3390/jcdd7030036 (PMC7570107; doi:10.3390/jcdd7030036)
Supplement: Supplementary file 1 [file jcdd-07-00036-s001.pdf]

Supplementary Materials

Transcatheter Versus Surgical Aortic Valve Replacement in Patients with Cardiac Surgery: Meta-Analysis and Systematic Review of the Literature

Azka Latif [1,*], Noman Lateef [1], Muhammad Junaid Ahsan [1], Vikas Kapoor [1], Rana Mohammad Usman [2], Stephen Cooper [1], Venkata Andukuri [1], Mohsin Mirza [1], Muhammad Zubair Ashfaq [1] and Rami Khouzam [3]

[1] Department of Internal Medicine, Creighton University, Omaha, NE 68124, USA; noman.mlateef@gmail.com (N.L.); junaidahsan333@gmail.com (M.J.A.); VikasKapoor@creighton.edu (V.K.); StephenCooper@creighton.edu (S.C.); VenkataAndukuri@creighton.edu (V.A.); Muhammad.Ashfaq@alegent.org (M.Z.A.); mohsin.mirza@creighton.edu (M.M.)

[2] Department of Internal Medicine, University of Tennessee, Memphis, TN 38152, USA; rusman@uthsc.edu

[3] Department of Cardiology, University of Tennessee, Memphis, TN 38152, USA; khouzamrami@yahoo.com

* Correspondence: azkalatif@creighton.edu; Tel.: +402-651-4961

Study name	Year	Statistics for each study				
		Odds ratio	Lower limit	Upper limit	Z-Value	p-Value
Chen et al.	2018	0.517	0.339	0.789	-3.058	0.002
Conte et al.	2016	0.284	0.108	0.746	-2.555	0.011
Greason et al.	2014	0.944	0.297	2.998	-0.098	0.922
Gupta et al.	2018	0.809	0.720	0.909	-3.561	0.000
Papadopoulos et al.	2014	0.487	0.042	5.599	-0.577	0.564
Wendet et al.	2015	2.036	0.499	8.314	0.991	0.322
Wilbring et al.	2013	1.461	0.433	4.932	0.611	0.542
		0.709	0.492	1.023	-1.840	0.066

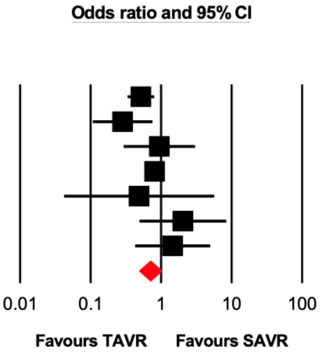

Supplementary Figure 1: Forest Plot showing comparing the risk of acute kidney injury between transcather versus surgical aortic valve replacement.

Study name	Time point	Difference in means	Standard error	Variance	Lower limit	Upper limit	Z-Value	p-Value
Chen et al.	2018	-193.200	6.722	45.186	-206.375	-180.025	-28.741	0.000
Papadopoulos et al.	2014	-226.500	20.742	430.225	-267.153	-185.847	-10.920	0.000
Wilbring et al.	2013	-97.700	4.904	24.051	-107.312	-88.088	-19.922	0.000
		-170.951	40.011	1600.908	-249.372	-92.530	-4.273	0.000

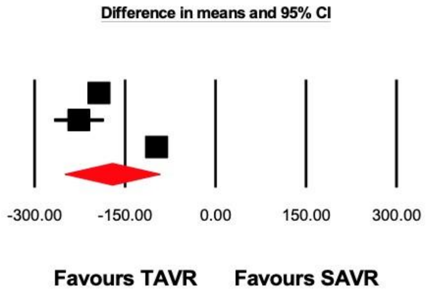

Supplementary Figure 2: Forest Plot showing comparing the procedural time between transcather versus surgical aortic valve replacement.

	Time point	Std diff in means	Standard error	Variance	Lower limit	Upper limit	Z-Value	p-Value
Chen et al.	2018	-0.940	0.093	0.009	-1.123	-0.756	-10.052	0.000
Conte et al.	2016	-0.460	0.135	0.018	-0.724	-0.196	-3.412	0.001
Gupta et al.	2018	-0.449	0.023	0.001	-0.494	-0.404	-19.531	0.000
Wilbring et al.	2013	-0.339	0.196	0.038	-0.722	0.045	-1.731	0.084
		-0.562	0.140	0.020	-0.837	-0.288	-4.013	0.000

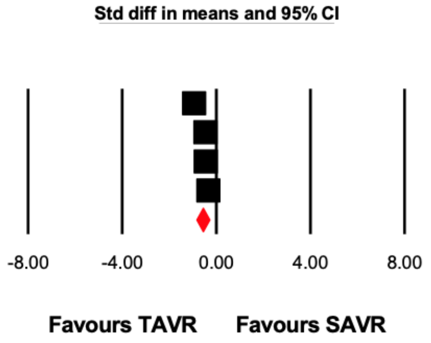

Supplementary Figure 3: Forest Plot showing comparing the length of hospital stay between transcather versus surgical aortic valve replacement.
